# Circulating Tumor DNA and [^18^F]FDG-PET for Early Response Assessment in Patients with Advanced NSCLC

**DOI:** 10.3390/diagnostics15030247

**Published:** 2025-01-22

**Authors:** Heidi Ryssel, Lise Barlebo Ahlborn, Danijela Dejanovic, Sune Hoegild Keller, Mette Pøhl, Olga Østrup, Annika Loft, Barbara Malene Fischer, Seppo Wang Langer, Andreas Kjaer, Tine Nøhr Christensen

**Affiliations:** 1 Department of Clinical Physiology and Nuclear Medicine, Rigshospitalet, Copenhagen University Hospital, 9 Blegdamsvej, 2100 Copenhagen, Denmark; heidi.ryssel@regionh.dk (H.R.); danijela.dejanovic@regionh.dk (D.D.); sune.hoegild.keller@regionh.dk (S.H.K.); annika.loft@regionh.dk (A.L.); malene.fischer@regionh.dk (B.M.F.); tine.noehr.christensen.02@regionh.dk (T.N.C.); 2 Department of Oncology, Rigshospitalet, Copenhagen University Hospital, 9 Blegdamsvej, 2100 Copenhagen, Denmark; mette.poehl@regionh.dk (M.P.); seppo.langer@regionh.dk (S.W.L.); 3 Cluster of Molecular Imaging, Copenhagen University, Panum Institution, 3 Blegdamsvej, 2200 Copenhagen, Denmark; 4 Department of Genomic Medicine, Rigshospitalet, 9 Blegdamsvej, 2100 Copenhagen, Denmark; lise.barlebo.ahlborn@regionh.dk (L.B.A.); osvarcova@gmail.com (O.Ø.); 5 Department of Clinical Medicine, University of Copenhagen, 2200 Copenhagen, Denmark; 6 School of Biomedical Engineering and Imaging Sciences, Kings College London, London WC2R 2LS, UK

**Keywords:** keyword 1, non-small cell lung cancer 2, circulating tumor DNA 3, [^18^F]FDG-PET/CT 4, early response evaluation 5, MTV 6, SUL 7, prognoses

## Abstract

**Background/Objectives**: Identifying treatment failure at earlier time points to could spare cancer patients from ineffective treatment and side effects. In this study, circulating tumor DNA (ctDNA) and [^18^F]FDG-PET/CT were investigated during the first cycle of anticancer therapy in patients with advanced non-small cell lung cancer (NSCLC) to explore their potential for early response evaluation. **Methods**: Patients with advanced NSCLC receiving first-line therapy with immune checkpoint inhibitors and/or chemotherapy were included. CtDNA and [^18^F]FDG-PET/CT assessments were conducted before treatment and at weeks 1 and 3 during the first cycle of therapy. ctDNA quantification was performed using a targeted next-generation sequencing (NGS) panel, and the least favorable change in any mutated allele frequency at a given time was used for analysis. [^18^F]FDG-PET/CT was quantified using sumSUL_peak_ and metabolic tumor volume (MTV_4.0_). Early changes in ctDNA levels and [^18^F]FDG-PET parameters were compared with final treatment response, measured by RECIST after 12 weeks, as well as progression-free survival and overall survival. **Results**: Of the sixteen included patients, eight were non-responders. ctDNA mutations were detected in baseline blood samples in eight patients. Changes in ctDNA level, MTV_4.0_, and sumSUL_peak_ at week 3 indicated response in 7 out of 8 patients, 13 out of 15 patients, and 9 out of 15 patients, respectively. At week 3, no false increases were seen with ctDNA and MTV_4.0_. **Conclusions**: These results suggest that early changes in ctDNA and [^18^F]FDG-PET/CT at 3 weeks of treatment could be used to early assess treatment response. Increased levels of ctDNA and MTV_4.0_ at week 3 were only observed in patients with treatment failure.

## 1. Introduction

Lung cancer is a severe disease with a poor prognosis and is a leading cause of cancer death [1]. A significant number of patients who progress on first-line treatment experience a decline in performance status, preventing them from receiving second- or third-line therapy [2]. Thus, time becomes critical, and early response evaluation is essential. An approach to response assessment is the use of biomarkers such as circulation tumor DNA (ctDNA) and 2-deoxy-2-[^18^F]fluoro-D-glucose ([^18^F]FDG) positron emission tomography (PET). They allow for monitoring of tumor cell death in real time and hence enable a more rapid assessment of treatment efficacy compared with conventional imaging.

ctDNA is small fragments of mutated DNA in plasma that is released from dying tumor cells. Due to the short half-life of ctDNA (15 min to 2.5 h), it has the potential for ongoing evaluation during treatment [3,4,5]. As ctDNA reflects active tumor cell death, it allows evaluation of tumor changes in hours rather than in weeks or months [6]. Previous studies have demonstrated the potential of ctDNA as a biomarker of cancer and its potential for response evaluation [4,7,8,9,10,11,12,13,14,15,16,17,18,19,20,21,22,23].

In patients with non-small cell lung cancer (NSCLC), several studies have shown associations between changes in ctDNA during treatment and final response, progression-free survival (PFS), and overall survival (OS) [7,8,9,10,11,12,13,14,15,16,17,18,20]. However, the majority of studies have focused on subgroups of patients with targetable genetic alterations (e.g., *EGFR* or *ALK* mutation) [16,17,18], and only few studies addressed the value of very early ctDNA changes during the first three weeks of treatment [7,8,9,10].

ctDNA analysis is routinely used for genotyping advanced NSCLC; however, early response assessment using plasma ctDNA is yet not well characterized. Various studies have used different definitions and thresholds of ctDNA dynamics, and the timing to assess ctDNA response has differed. A more standardized approach is needed to move from clinical validity to clinical utility [24].

[^18^F]FDG-PET is a surrogate marker of glucose metabolism, and it is considered a standard procedure for the clinical staging of NSCLC [25,26,27]. Studies have implied that [^18^F]FDG-PET/CT may be used for early response evaluation and that loss of viable cancer cells during therapy will cause a decline in [^18^F]FDG-uptake prior to structural changes becoming visible at CT [28,29,30,31]. However, it has been suggested that the results may be unstable during the first weeks after treatment, and the optimal timing of early response evaluation with [^18^F]FDG-PET/CT is still debatable [29,31,32,33,34].

Despite ctDNA and [^18^F]FDG-PET/CT both being promising modalities, studies that combine ctDNA and [^18^F]FDG-PET for response evaluation in NSCLC are limited. Baseline ctDNA has shown correlation with various [^18^F]FDG-PET parameters in both early stage disease [35,36,37] as well as advanced NSCLC [38,39,40]. To our knowledge, only one previous study has investigated ctDNA and [^18^F]FDG-PET changes during or after treatment within the same patient cohort, finding that both were prognostic for OS and PFS after 6 weeks of treatment [19].

The combination of ctDNA and [^18^F]FDG-PET/CT may provide us with information of both molecular and metabolic changes and hereby improve early response evaluation. In this study, we assessed the utility of ctDNA and [^18^F]FDG-PET/CT in early response evaluation in patients with advanced NSCLC to detect treatment failure one week and three weeks after treatment initiation.

## 2. Materials and Methods

### 2.1. Study Design

In this prospective observational study, patients with treatment-naïve advanced NSCLC referred for palliative treatment was eligible. Patients were recruited from the Dept. of Oncology, Rigshospitalet, Denmark from April 2018 to February 2020.

The trial was approved by the local ethics committee (H-18042903 and H-17024315) and The Danish Data Protection Agency (RH-2015-04, I-suite03 605). All patients provided informed consent.

### 2.2. Participants

This study enrolled patients with newly diagnosed advanced NSCLC (stage IIIB–IV) referred for first-line treatment with chemotherapy and/or immune checkpoint inhibitors (ICIs). Exclusion criteria included synchronous cancer, pregnancy, chronic inflammatory disease, performance status (according to the Eastern Cooperative Oncology Group Performance Status—ECOG score) of > 2 [41], or previously identical antineoplastic treatment.

Staging was performed according to the 8th edition of TNM (the American Joint Committee on Cancer and Union for International Cancer Control TNM staging) [3].

[^18^F]FDG-PET/CT scans and blood samples were planned before treatment initiation (baseline), at week 1 after treatment, at week 3 (before the 2nd cycle of treatment), and at the time of the final CT evaluation—approximately week 13.

Follow-up was ended in December 2023.

### 2.3. Blood Sampling

Consecutive blood samples were collected in cell-stabilizing BCT-tubes (Streck Laboratories, La Vista, NE, USA, Cat# 218997). At each timepoint, two BCT-tubes were collected. Blood samples were taken prior to [^18^F]FDG-PET/CT scan or chemotherapy. Blood samples at week 1 were performed only in patients who were scheduled for examination at the hospital (either blood samples or [^18^F]FDG-PET/CT-scan). Plasma was isolated by two-step centrifugation: first at 2000× *g* for 10 min at 20 °C and subsequently at 16,000× *g* for 10 min at 20 °C. Cell-free DNA was extracted from 8 mL plasma using the QIAsymphony Circulating DNA Kit (Qiagen, Germantown, MD, USA) according to the manufacturer’s instructions and quantified using a dsDNA HS Assay Kit on the Qubit Fluorometer (Thermo Fisher Scientific, Waltham, MA, USA).

### 2.4. ctDNA Analysis

Cell-free DNA samples were sequenced by using the TruSight Oncology (TSO) 500 HT gene panel (Illumina, San Dieg, CA, USA). TSO 500 is a targeted next-generation sequencing (NGS) panel targeting 523 genes. In brief, DNA libraries were prepared from a minimum of 10 ng cell-free DNA and hybridized using the TSO500 HT assay. The libraries were sequenced on the NovaSeq6000 platform to a minimum median coverage of 600×. Sequencing reads were mapped to the hg38/GRCh38 human reference genome using BWA-MEM v0.7.12 software, and somatic mutations were called using GATK Mutect2 Best Practices guidelines including removal of polymorphisms present in >1% of the general population (gnomAD). Mutations were inspected using QIAGEN Clinical Insight (QCI) Interpret Translational software (version v.8.0.), and each mutation was manually inspected in the raw sequencing reads using the Integrative Genomics Viewer (IGV).

Pathogenic or likely pathogenic somatic mutations, including nonsense, frameshift, missense, and splice site alterations (±2 bp) in cancer-related genes, were reported down to a mutant allele frequency (MAF) of 5%. However, for cancer hotspot mutations or well-described tumor alterations, we included these alterations down to a ~1% MAF. All mutations reported in the baseline ctDNA sample were manually inspected in the sequencing reads from subsequent ctDNA samples to detect mutations at exceptionally low MAFs (<1%). All mutations were checked in the raw sequencing reads. Mutations in ctDNA with an MAF of 40–100% were considered germline mutations and excluded if they shoved stable values in all consecutive samples.

### 2.5. Image Acquisition and Analysis

The [^18^F]FDG-PET/CT scans were performed at Rigshospitalet on a Siemens Biograph TruePoint, Siemens Biograph mCT, or Siemens Biograph Vision 600. Static regional images were obtained from the skull base to the mid-thighs. The CT was performed as a low-dose scan without contrast. Image acquisition was started approximately 60 min after an injection of 4 MBq (megabecquerels)/kg [^18^F]FDG. Patients fasted at least 4 h prior to the injection, and they were asked to rest after the injection. Consecutive [^18^F]FDG-PET/CT scans on the same patient were performed on identical PET/CT scanner models and preferably on the exact same PET/CT scanner. The [^18^F]FDG-PET scans were reconstructed using an Ordered-Subset Expectation Maximization (OSEM) with point-spread-function modeling with either 3 iterations and 21 subsets for TruePoint, 2 iterations, 21 subsets, and Time-of-Flight for mCT, or 4 iterations, 5 subsets, and Time-of-Flight for Vision) and a Gaussian post-reconstruction filter of 2 mm full width at half maximum.

[^18^F]FDG-PET/CT was analyzed using syngo.via (Siemens Healthineers, Erlangen, Germany). For research purposes, up to five malignant lesions were identified by an experienced nuclear medicine physician. The [^18^F]FDG uptake was quantified using a standardized uptake value corrected for lean body mass (SUL), as suggested in the PERCIST criteria [28]. In the malignant lesions, a volume of interest (VOI) was delineated using 50% of SUL_max_ as the threshold, as suggested for tumors with heterogenous uptake by the EANM procedural guidelines [42]. It was noticed that this VOI in some cases did not correspond visually with the lesion; therefore, an additional VOI with an absolute threshold of SUL_max_ of > 4.0 was generated post hoc. This segmentation method was chosen based on a known superiority in patients with lymphoma [43]. Within both VOIs, metabolic tumor volume (MTV), maximum SUL (SUL_max_), average SUL (SUL_mean_), peak SUL (SUL_peak_; SUL_mean_ in a sphere with a volume of 1 cm^3^, placed in the VOI to maximize the value), and total lesion glycolysis (TLG = MTV x SUL_mean_) were measured. High-fidelity SUL measurements from quantitatively accurate PET scans were ensured through a rigorous cross-calibration procedure as detailed in [44].

### 2.6. Endpoints

Measurements from the ctDNA analysis and [^18^F]FDG-PET/CT were compared with the final response, PFS, and OS. Response was assessed according to RECIST v. 1.1. [45] on CT following three months of treatment. Patients were categorized as responders if they obtained disease control (stable disease (SD), partial response (PR), or complete response (CR)) or as non-responders if they progressed during treatment (progressive disease (PD)). OS was defined as the time from baseline study investigations to death from any cause. PFS was defined as the time from baseline study investigations to progression or death. The date of progression was defined as the first investigation implying progression, even if further investigations were needed to confirm progression, i.e., in the case of pseudo-progression.

### 2.7. Statistical Methods

Statistical analyses were performed using IBM SPSS (IBM Corp., Armonk, NY, USA) Statistics version 29.0.1.0. Figures were created in IBM SPSS Statistics 29.0.1.0 and rStudio 2024.04.2. For clinical relevance and simplicity, the least favorable change in any MAF in ctDNA at a given time was used for analysis, i.e., the largest increase or smallest decrease from the baseline. Appearance of new detectable mutations were considered as relapse and quantified as an 100% increase. For the quantitative analyses of the [^18^F]FDG-PET parameters, the highest SUL_max_ (maxSUL_max_), the highest SUL_peak_ (maxSUL_peak_), the sum of SUL_peak_ (sumSUL_peak_), the sum of MTV (totalMTV_50%_ and totalMTV_4.0_), the sum of TLG (totalTLG_50%_ and totalTLG_4.0_), and the average of SULmean in all VOIs (totalSUL_mean50%_ and totalSUL_mean4.0_ calculated as totalTLG/totalMTV) were used. Relative changes of ctDNA and [^18^F]FDG-PET-parameters from baseline to week 1, week 3, and week 13 were used for the analyses. ctDNA and [^18^F]FDG-PET parameters changes were dichotomized by increasing and decreasing values. The association between baseline [^18^F]FDG-PET parameters and baseline ctDNA (detectable vs. non-detectable) was calculated using an independent *t*-test or Welch’s test. Correlations were measured using Pearson’s correlation. Kaplan−Meier graphs were created. A *p*-value of < 0.05 was considered significant.

## 3. Results

### 3.1. Participants

**Figure 1:**
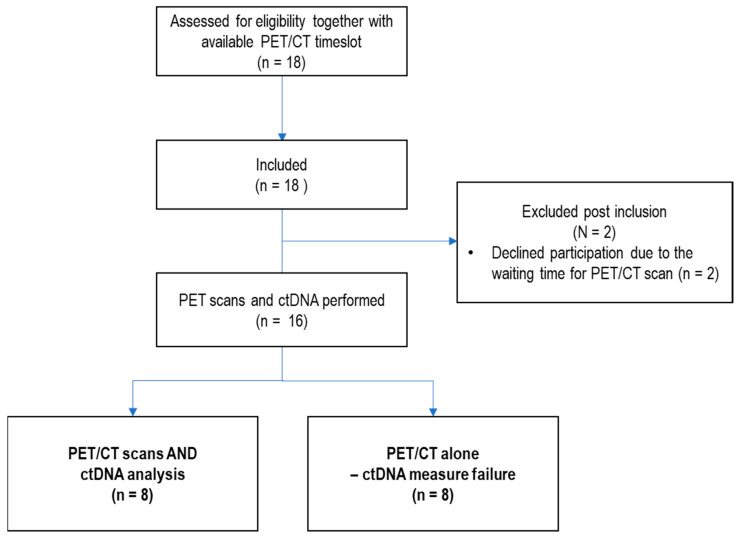
Flowchart displaying patient screening, patient inclusion, and exclusions in the study.

Eighteen patients with newly diagnosed NSCLC were included in the study. Two patients withdrew their consent due to a delay of the first [^18^F]FDG-PET/CT scan; thus, sixteen patients were enrolled in the study (Figure 1). Nine patients received chemotherapy, and seven patient received immune checkpoint inhibitors—either in combination with chemotherapy or as monotherapy. One patient (ID 13), initially evaluated to be unfit for radiotherapy, received curatively intended radiotherapy after the final response evaluation. Another patient (ID 10) received palliative radiotherapy for a bone metastasis after enrollment. Patient characteristics are summarized in Table 1.

**Figure 2:**
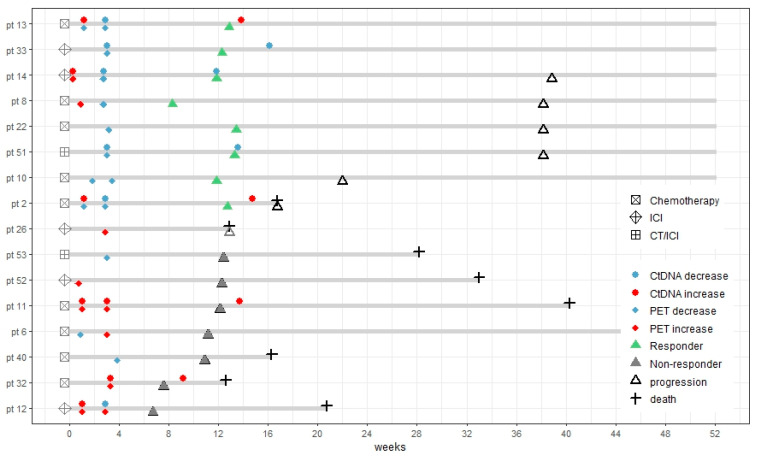
Swimmer plot showing the timelines for all study patients during the first year after inclusion. Each patient is listed individually by their ID numbers (pt = ID). Patients were arranged by final response: response versus non-response. Second, patients were ranged by progression-free survival. Initial treatment, timing, and results from ctDNA (measured as the least favorable change in mutated allele frequency) and [^18^F]FDG-PET/CT (measured as the change in totalMTV_4.0_) and patient outcomes are shown. ctDNA and totalMTV_4.0_ stratified by increase and decrease in the first week after treatment initiation did not show any convincing association with final response. Increasing levels of ctDNA or totalMTV_4.0_ at week 3 were observed exclusively in patients with progression at the final response evaluation. At the time of the final response evaluation, four patients had an increased ctDNA level; however, two patients were responders on the evaluation scan. Both patients had possible explanations for the increase: one (ID 2) died shortly after due to massive progression of the liver metastasis, and the other patient (ID 13) received definitive radiotherapy of 66 Gy after the final response evaluation; therefore, an underlying progression could not be excluded in this patient as well. The patient (ID 6) was alive after 12 months despite being a non-responder on the evaluation scan. This was due to a second-line therapy with a durable tumor response. ICI—immune checkpoint inhibitor; CT/ICI—combination of chemotherapy and immune checkpoint inhibitors.

The final response evaluation was performed with CT a median of 12 weeks after the start of the treatment. In one patient, progression was not confirmed by CT though the patient (ID 26) showed clinical signs of progression and died 3 months after inclusion. A total of eight patients were responders, and eight patients were non-responders. The timeline for each patient is illustrated in Figure 2, including the timing and results of ctDNA and [^18^F]FDG-PET/CT and clinical outcomes.

### 3.2. ctDNA

In total, 59 blood samples were collected. Baseline ctDNA was detectable in only eight patients due to a too low cell-free DNA concentration in the samples (n = 6); sufficient cell-free DNA concentration but non-detectable ctDNA mutations in the TSO500 analysis (n = 1); or missing baseline sample collection (n = 1). Patients were excluded from further ctDNA analysis if baseline ctDNA was undetectable. ctDNA at week 13 was excluded in one patient due to the initiation of a 2nd-line treatment before ctDNA was collected. ctDNA was available from five patients at week 1 (median: 7 days after baseline; inter quartile range (IQR): 7–8 days), eight patients at week 3 (median: 21 days; IQR: 20–21 days), and seven patients at week 13 (median: 13.7 weeks; IQR: 13–14 weeks). ctDNA analysis revealed between 1 and 6 pathogenic mutations for each patient in 19 different genes. *TP53* was the most frequently affected gene, and mutations in *TP53* was found in seven out of eight patients at baseline. Other affected genes found in more than one patient was *CHEK2* (n = 2) and *KRAS* (n = 2) (Supplementary Figure S1 and Supplementary Table S1). The clinical characteristics did not differ in patients with detectable ctDNA compared to without detectable ctDNA (Table 1).

### 3.3. [^18^F]FDG-PET Parameters

A total of 40 [^18^F]FDG-PET/CTs were completed in this study. All patients completed the baseline scan; nine patients completed the scan at week 1 (median: day 7; IQR: 6–8); fifteen patients completed the scan at week 3 (median day 21; IQR: 20–22 days). Image acquisition was started at a median of 61 min (IQR: 59–66 min) after an injection of 226–454 MBq [^18^F]FDG.

**Figure 3:**
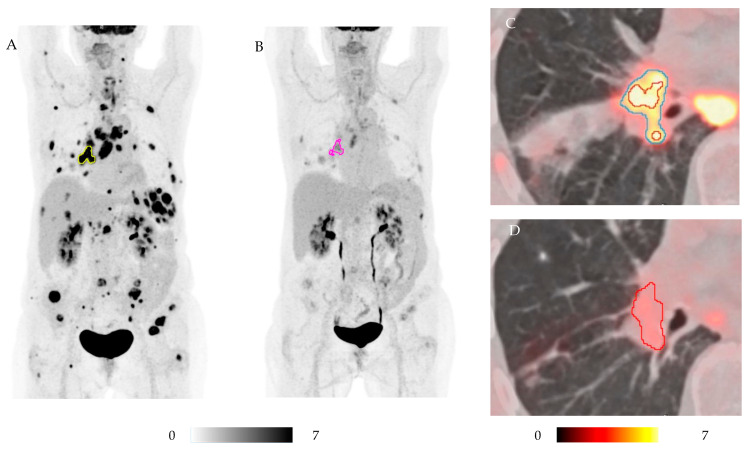
[^18^F]FDG-PET/CT at baseline (**A**,**C**) and at week 3 (**B**,**D**) in a patient with partial response to chemotherapy and immune checkpoint inhibitors. In this patient, ctDNA was non-detectable at week 3. Visually, the number of detectable lesions and the intensity of the [^18^F]FDG uptake reduced substantially (multi-image projections at baseline with MTV_50%_ delineated in the primary tumor with yellow (**A**) and pink (**B**)), and all [^18^F]FDG-PET parameters, but MTV_50%_, decreased. As seen from the transverse projection of the primary tumor in the left hilus (**C**,**D**), MTV_50%_ (delineated with the red line) increased by 60% from 3.84 ccm to 6.18 ccm. MTV_4.0_ (delineated with the blue line) was initially larger than MTV_50%_, but not detectable at week 3. This [^18^F]FDG-PET/CT illustrated that the [^18^F]FDG-PET parameter MTV_50%_ did not always correspond with the visual impression or other [^18^F]FDG-PET parameters and that it should be used with caution.

Most [^18^F]FDG-PET parameters correlated strongly or very strongly with each other; however, totalMTV_50%_ did not show significant correlation with any SUL parameter, and totalTLG_50%_ correlated only moderately with the SUL parameters. In addition, in some cases, MTV_50%_ did not correspond with the visual evaluation. In particular, MTV_50%_ seemed misleading in patients with high SUL_max_ values and in patients with large changes in SUL_max_, as illustrated in Figure 3. This paper focuses on totalMTV_4.0_ and sumSUL_peak_. All [^18^F]FDG-PET parameters are available in Supplementary Table S2.

### 3.4. Association Between ctDNA and [^18^F]FDG-PET/CT

The [^18^F]FDG-PET parameters did not significantly differ in patients with detectable baseline ctDNA compared with those in patients in whom ctDNA was not detectable. At week 3, the ctDNA changes were significantly correlated with changes in sumSUL_peak_ (*p* = 0.005; r = 0.870; n = 8) and totalMTV_4.0_ (*p* = 0.027; r = 0.765; n = 8). The highest MAF at baseline and ctDNA change at week 1 did not correlate significantly with any [^18^F]FDG-PET parameter.

### 3.5. Association with Final Response

**Figure 4:**
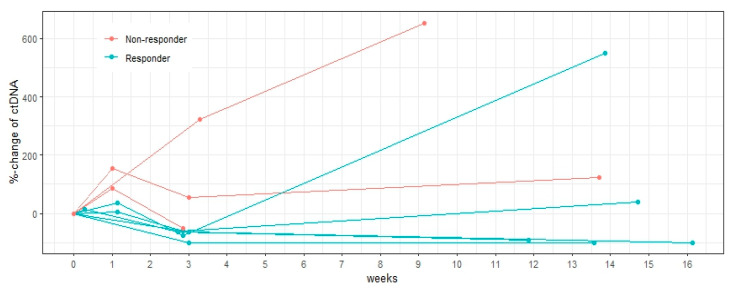
Relative changes in ctDNA from baseline until final response evaluation, stratified by final response. The least favorable changes in ctDNA (mutant allele frequency) are displayed for each patient at week 1, week 3, and week 13. At week 1, ctDNA increased in all patients; however, larger increases were seen in non-responders than in responders. At week 3, six patients had relative decreases in ctDNA, and the largest reductions were found in responders.

**Figure 5:**
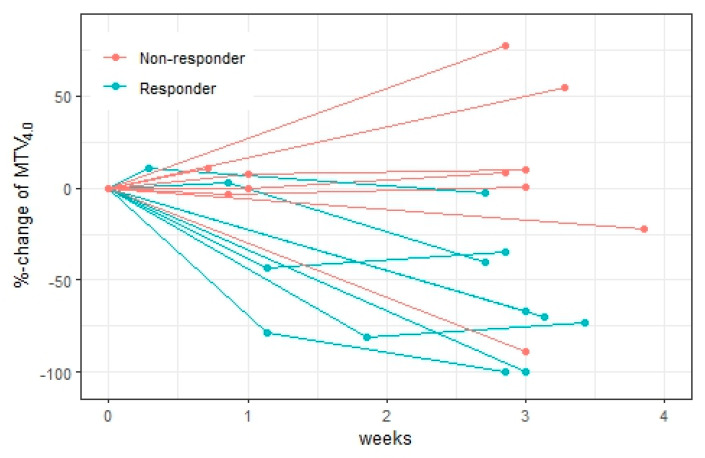
Relative changes in totalMTV_4.0_ during the first cycle of treatment. Patients were stratified by final response. In week 1, the totalMTV_4.0_ changes agreed with final response in six of nine patients, but false increases in two responders and a false decrease in one non-responder were noticed. In week 3, the relative changes in totalMTV_4.0_ stratified the responders from non-responders in 13 of 15 patients. One non-responder had an 89% decrease in totalMTV_4.0_ (sumSUL_peak_ decreased by 39%) in week 3. At the final response evaluation, this patient was suspected of having pseudo-progression due to the growth of a single lymph, and progression was confirmed 2 months later. This lymph node was not detected by [^18^F]FDG-PET/CT even upon retrospective review. Unfortunately, ctDNA was not available from this patient.

**Figure 6:**
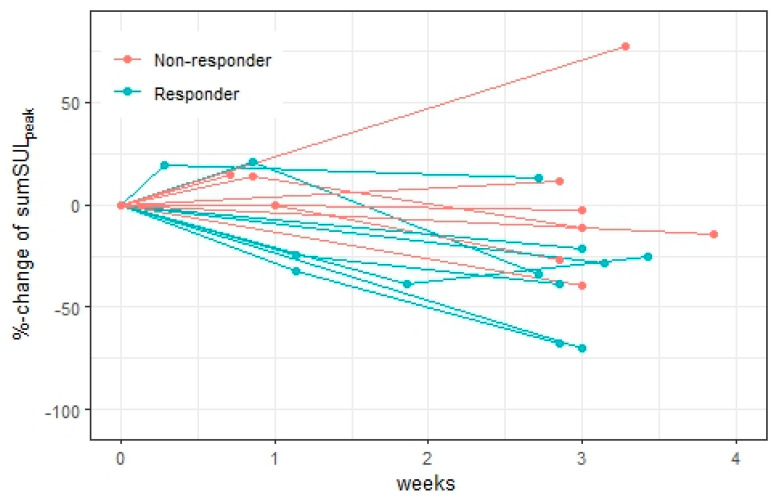
Relative changes in sumSUL_peak_ during the first cycle of treatment, stratified by final response. The relative changes in sumSUL_peak_ did not seem to agree with the final response at week 1 or week 3; however, in many patients, the relative changes in sumSUL_peak_ were small.

The relative changes in ctDNA, totalMTV_4.0_, and sumSUL_peak_ stratified by final response are illustrated in Figure 4, Figure 5 and Figure 6.

At week 1, ctDNA increased from baseline in all patients (n = 5) with a more pronounced increase in non-responders (87–156%) compared to in responders (3–38%). The changes in totalMTV_4.0_ and sumSUL_peak_ predicted the final response in six and five of nine patients, respectively. However, in many patients, the PET parameter changes were small (<25%), and of note, only minor changes were seen in all patients in whom totalMTV_4.0_ and sumSUL_peak_ disagreed with the final response.

At week 3, six patients had relative decreases in ctDNA, and the ctDNA changes correctly predicted the final response in seven out of eight patients. The largest ctDNA decreases were seen in the responders (>56%) whereas the non-responders had either an increase in ctDNA or a decrease of less than 50%.

Changes in totalMTV_4.0_ were associated with final response in 13 out of 15 patients, and changes in sumSUL_peak_ were associated with final response in 9 of 15 patients. totalMTV_4.0_ decreased in all responders, but decreases were also seen in two non-responders (22% and 89%). Diagnostic accuracy metrics for ctDNA and all [^18^F]FDG-PET parameters are available in Supplementary Table S3.

ctDNA and totalMTV_4.0_ responses at week 3 agreed in seven out of eight patients. In the patient (ID 12), ctDNA decreased whereas totalMTV_4.0_ correctly identified the patient as a non-responder.

ctDNA levels at week 13 aligned with the final response in five out of seven patients. Of note, one patient (ID 2), evaluated (week 13) as having PR but with an increased ctDNA level at week 13, died after 28 days due to massive progression. Another patient (ID 13), also evaluated with PR and an increase in ctDNA (more than 550% from the baseline level) at week 13, received radiotherapy (66 Gy delivered as 2.0 Gy in 33 fractions) following the final response evaluation. Radiotherapy was not initiated until after final response evaluation and therefore not the reason for the increasing ctDNA observed in the patient (ID 13). In this case, presumptive progression, indicated by the increase in ctDNA, could not be ruled out.

### 3.6. Prognostic Value of ctDNA and [^18^F]FDG-PET/CT

**Figure 7:**
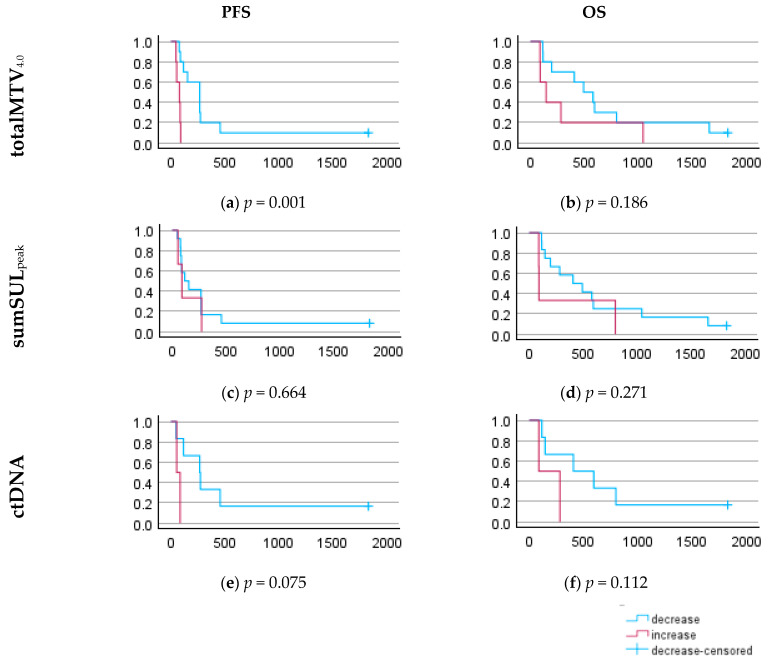
Progression-free survival (PFS) and overall survival (OS) stratified by patients with increasing and decreasing values at week 3, indicated by Kaplan−Meier curves. PFS was stratified by the following parameters: (**a**) totalMTV_4.0_; (**c**) sumSUL_peak_; (**e**) ctDNA. OS was stratified by the following parameters: (**b**) totalMTV_4.0_; (**d**) sumSUL_peak_; (**f**) ctDNA.

At the end of the follow-up, 15 patients had died from progressive disease. One patient, without progression, was censored after 5 years. The median PFS was 3 months (IQR: 3–9 months), and the median OS was 11 months (IQR: 4–25 months). Patients with decreasing ctDNA at week 3 tended to have longer PFS and OS than patients with increasing ctDNA. Patients with a decrease in totalMTV_4.0_ had significantly longer PFS than patients with an increase in totalMTV_4.0_ (Figure 7).

## 4. Discussion

This study highlights associations between early changes in ctDNA level and [^18^F]FDG-PET parameters and treatment response in patients with advanced NSCLC. An increase in ctDNA level or MTV_4.0_ at week 3 after treatment initiation was seen only in patients with disease progression. Patients with decreasing ctDNA or MTV_4.0_ tended to have longer PFS and OS. However, both ctDNA and MTV_4.0_ showed false positive increases at week 1, indicating the importance of timing. Nevertheless, ctDNA levels increased more in non-responders than in responders at week 1, suggesting ctDNA at week 1 has the potential that should be further explored.

### 4.1. ctDNA

In consistence with our findings, previous studies on NSCLC patients have shown associations between early changes in either ctDNA or [^18^F]FDG-PET/CT within the first months after the initiation of non-targeted treatment with treatment response, PFS, and OS [7,8,9,10,11,12,14,15,29,30,46,47].

However, different studies have processed and reported ctDNA data differently, which make comparisons less transient, and the optimal way to report ctDNA has yet to be defined [24].

In our study, the entire TSO 500 panel was performed at all follow-up investigations, and the least favorable change in all allele frequencies at any given time was reported. This method was chosen to reduce the risk of overlooking new mutations or new dominant clones that could be crucial for detection of early progression and to mimic conventional response evaluation. However, other studies have reported only ctDNA mutations confirmed in tissue samples [10] or only “the highest allele frequency” were followed over time [7,8]. Despite these approaches seeming clearly defined, there is a risk of overlooking new mutations or newly increasing subclones that may be of clinical relevance.

Despite the use of different methods for processing ctDNA results, outcomes have been comparable, including a few previous studies that have focused on ctDNA changes during the first cycle of therapy and their association with outcomes [7,8,9,10]. One study identified week 2 to be a significant time point for evaluating ctDNA changes but also described a temporary increase at week 1 among responders [10]. In the current study, we observed increasing ctDNA levels in all patients at week 1 after treatment initiation, though more pronounced in non-responders. A previous study used a cutoff of a 50% decrease to stratify ctDNA response from non-response at week 3 [8]. If using this cutoff at week 1 in the current study, responders and non-responders would be well differentiated. The potential of ctDNA for very early response evaluation at week 1 is an interesting observation to be further explored in future studies.

### 4.2. [^18^F]FDG-PET/CT

Several studies have demonstrated the potential of early response evaluation with [^18^F]FDG-PET/CT after several treatment regimes, including chemotherapy, targeted therapy, radiotherapy, and ICI [29,30,46,47,48,49,50,51]. The above studies vary in the timing of the [^18^F]FDG-PET/CT, definition of PET parameters, and treatment; however, in general, the studies have appointed MTV or TLG to be better predictors and prognosticators than SUL or SUV [30,49,50,51]. This agrees with our results that MTV_4.0_ changes predict final response and prognosis better than sumSUL_peak_.

In the current study, we noticed that the method of segmentation is crucial for the results. MTV segmented by 50% of SUL_peak_ did not always align with the visual impression of tumor extent, particularly in patients with high SUL_peak_. In patients with a large reduction in SUL_peak_ and thus a substantial reduction in the absolute value used for segmentation, the change in MTV_50%_ seemed misleading. In the current study, the changes in MTV_4.0_ aligned with the final response in 13 out of 15 patients and was significantly associated with PFS, whereas MTV_50%_ aligned with the final response in only 11 out of 15 patients with both false increases and false decreases. Using an SUV of _4.0_ for segmentation has been recommended based on a study comparing several segmentation methods in patients with diffuse large B-cell lymphoma [43]. Recently, Tricarico et al. [52] proved MTV_4.0_ to be superior to SUV_max_, TLG, and PERCIST in patients with NSCLC, measured 6–8 weeks after the initiation of ICI. Tricarico et al. even called MTV_4.0_ a game changer. Our results are in accordance with the results by Tricarico et al., and the current results additionally suggest that MTV_4.0_ has prognostic value as early as 3 weeks after treatment initiation.

### 4.3. Relation Between ctDNA and [^18^F]FDG-PET

Larger studies have found a moderate correlation between absolute values of [^18^F]FDG-PET parameters (SUV_max_, MTV, and TLG) and ctDNA [19,35,36,37,38,39,40]. To our knowledge, only one previous study has compared ctDNA and [^18^F]FDG-PET/CT changes with response evaluation [19] and found a correlation between MAF at week 6 and [^18^F]FDG-PET parameter changes after 6 weeks (two treatment cycles). Clearance of ctDNA was significantly associated with final response and OS, while MTV change was significantly associated with final response. The study was limited to a predefined panel (*EGFR*, *KRAS*, *BRAF*, *PIK3CA*, and *TP53*).

To our knowledge, the current study is the first to focus on the relationship and changes in ctDNA and [^18^F]FDG-PET parameters during one cycle of chemotherapy. Our study is strengthened by the use of a wide and standardized ctDNA panel covering 523 genes; a very homogeneous patient group; longitudinal data collection visualize the development at very early time points; and paired measurements with ctDNA and [^18^F]FDG-PET in each patient.

Limitations of this study include the number of patients, the sensitivity of the ctDNA detection, and the less pronounced changes in the biomarkers at an early stage in treatment. Recruitment and logistics of scheduling several [^18^F]FDG-PET/CT scans within a few days are known to be difficult [29], and the main limitation of this study is the limited number of patients. This was due to logistic challenges with the PET/CT scanners and the tight time schedule—from patient inclusion to performing the first PET/CT before treatment initiation.

In this study, the detection rate of ctDNA was only 50%. In comparative studies, detection rates were 50–81% [7,10,11,12,19,21,38,40,53]. Due to the limited number of patients in this study, the ctDNA and [^18^F]FDG-PET responses were dichotomized by decreased and increased parameter. According to the PERCIST criteria, changes in SUL_peak_ must be larger than 30% to categorize the change as response or progression [28]. Although lower changes of 20–25% in TLG and SUV_max_ has been suggested [48,49], response categories have not been established for other [^18^F]FDG-PET-parameters or for ctDNA. The changes in MTV_4.0_ and sumSUL_peak_ were small (<22%) in 5–6 patients at week 1 and week 3. If changes of <30%, or even <25%, had been categorized as “no change”, accuracy would have been higher for all parameters.

Making associations between ctDNA and [^18^F]FDG-PET/CT can be challenging as ctDNA is a marker of cancer cell death and its catena shedding is impacted not only by tumor burden but also by anatomic location and genomic subtype. In contrast, PET is a (unspecific) marker of metabolism [39]. The discrepancy in the dynamics of these modalities may, therefore, not reflect false results in all cases, but rather different biological tumor behaviors. Patients with divergent ctDNA and [18F]FDG-PET dynamics may need special attention; however, larger studies are needed to further investigate these associations.

From a clinical perspective, the use of ctDNA and [^18^F]FDG-PET/CT scans in early response evaluation opens the possibility of detecting treatment failure earlier, allowing timely treatment changes. A combination of ctDNA and [^18^F]FDG-PET/CT could contribute to a more reliable early response evaluation than ctDNA as [^18^F]FDG-PET/CT is more robust in providing an answer. ctDNA and [^18^F]FDG-PET/CT are easily implemented as they are already part of the clinical routine and both methods are minimally invasive and carry minimal risk to the patients. A biomarker of progressive disease with high specificity could be an important tool for clinicians.

## 5. Conclusions

ctDNA and MTV_4.0_ measured by [^18^F]FDG-PET/CT at week 3 have potential for early evaluation of treatment response. A key finding in this study was that ctDNA and MTV_4.0_ increases at week 3 were observed only in non-responders. Very early response evaluation at week 1 revealed false increases in both ctDNA and MTV_4.0_ and should be interpreted with caution. These results suggest the need for investigations in a larger prospective study. Ongoing research in the field of biomarkers for early treatment assessment is highly relevant for patients with NSCLC to spare future patients from unnecessary treatment.

## Figures and Tables

**Table 1:** Patient characteristics showing demography data all patients, patients with available ctDNA, and patients with undetectable ctDNA. No significant differences were found between the two groups with or without ctDNA. ICI—immune checkpoint inhibitor; CT/ICI—combination of chemotherapy and immune checkpoint inhibitors; PS—ECOG performance status; NA—not available.

	All Patients n = 16	Patients with ctDNA n = 8	Patients with ctDNA NA n = 8
Mean age (range)	69 years (52–80)	68 years (52–80)	70 years (54–79)
Gender (female)	8	4	4
Tobacco use			
Ever (n)	4	2	2
Former (n)	11	6	5
Never (n)	1	0	1
Histology			
Squamous (n)	5	3	2
Non-squamous (n)	11	5	6
Stage			
IIIB (n)	7	4	3
IV (n)	9	4	5
PDL-1 expression			
>50% (n)	5	3	2
1–50% (n)	9	5	4
<1% (n)	1	0	1
Unknown (n)	1	0	1
PS			
PS 0 (n)	8	3	5
PS 1 (n)	6	4	2
PS 2 (n)	2	1	1
Treatment			
Chemotherapy (n)	9	4	5
ICI (n)	5	3	2
CT/ICI (n)	2	1	1

## Data Availability

All data supporting the results can be found in Supplementary Tables S1 and S2.

## Supplementary Materials

The following supporting information can be downloaded at: https://www.mdpi.com/article/10.3390/diagnostics15030247/s1, Figure S1: ctDNA development; Table S1: ctDNA data; Table S2: Dataset including therapy, outcome, and absolute values of all PET parameters; Table S3: Association between changes in PET parameters (decrease vs. increase) and final response (PR + SD vs. PD).
