# Supplementary material for: Circulating Tumor DNA and [18F]FDG-PET for Early Response Assessment in Patients with Advanced NSCLC
Source: Diagnostics (Basel). 2025 Jan 22;15(3):247. doi: 10.3390/diagnostics15030247 (PMC11817175; doi:10.3390/diagnostics15030247)
Supplement: Supplementary file 1 [file diagnostics-15-00247-s001.zip › diagnostics-3365096-supplementary.pdf]

Supplementary

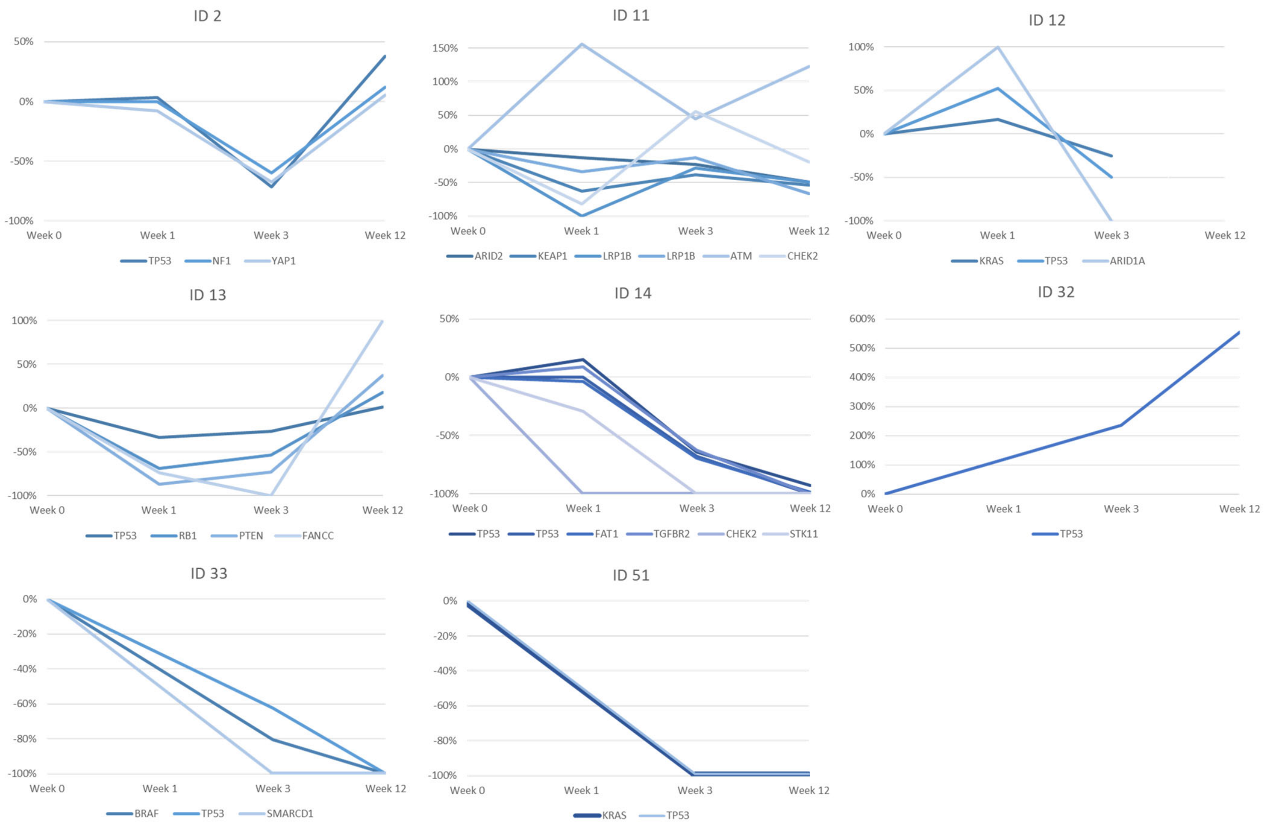

Supplementary Figure S1: CtDNA development. Patients with available ctDNA are each listed in separate figures with their ID number. Figures displays the development of the mutant allele frequency of each detected mutation from inclusion at baseline (at week 0) until evaluation (at week 12). The y-axis displays ctDNA change from baseline.

Supplementary Table S1: CtDNA data

ID			Variant		Baseline		Week 1		Week 3		Week 12		
	**Gene**	**Transcript**	**Coding**	**Protein**	**Frequency (%)**	**Reads (x)**	**Frequency (%)**	**Reads (x)**	**Frequency (%)**	**Reads (x)**	**Frequency (%)**	**Reads (x)**	**Note**
**2**	*TP53*	NM_000546.6	c.517G>T	p.V173L	29.00	1122	30.00	1666	8.28	978	40.00	847	
	NF1	NM_000267.3	c.7855_7861delTATGAAT	p.Y2619fs*2	17.00	795	17.00	1656	6.86	846	19.00	1961	
	YAP1	NM_006106.5	c.832G>T	p.E278*	37.00	1581	34.00	2295	12.00	1210	39.00	1820	
**11**	*ARID2*	NM_152641.4	c.3888delG	p.R1297fs*25	5.30	1334	4.60	263	4.10	1622	2.70	1278	
	KEAP1	NM_012289.4	c.1528delG	p.A510fs*22	12.00	1098	4.50	221	7.40	1100	5.60	864	
	LRP1B	NM_018557.3	c.8958C>G	p.Y2986*	2.37	1488	ND		1.70	1642	1.20	1401	
	LRP1B	NM_018557.3	c.12975C>A	p.C4325*	8.40	678	5.60	195	7.30	886	2.80	812	
	ATM	NM_000051.4	c.6056A>G	p.Y2019C	0.90	1059	2.30	258	1.30	1295	2.00	2000	
	CHEK2	NM_001005735.2	c.1245_1246delinsTG	p.K416E	2.50	1192	0.47	3779	3.90	1463	2.01	4385	
**12**	*KRAS*	NM_004985.5	c.34G>T	p.G12C	20.00	1291	26.00	415	10.00	1103	**		
	TP53	NM_000546.6	c.818G>T	p.R273L	17.00	1520	28.00	438	8.56	1461	**		
	ARID1A	NM_006015.6	c.1582C>T	p.Q528*	1.07	1781	2.00	410	ND	3950	**		
**13**	*TP53*	NM_000546.6	c.524G>A	p.R175H	5.54	523	0.24	4154	ND	1247	6.00	616	
	RB1	NM_000321.3	c.1243_1245delinsT	p.I415Sfs*12	2.06	339	ND		ND	840	5.10	594	
	PTEN	NM_000314.8	c.850G>T	p.E284*	2.00	514	1.00	4150	0.50	737	5.5	361	
	FANCC	NM_000136.3	c.1225_1229delGAGCA	p.E409fs*24	0.8	1158	1.10	4246	ND	1421	5.2	808	
**14**	*TP53*	NM_000546.6	c.659A>G	p.Y220C	13.00	2184	15.00	1797	4.68	705	0.89	4948	
	TP53	NM_000546.6	c.745A>T	p.R249W	13.00	1906	13.00	1837	4.19	835	ND		
	FAT1	NM_005245.4	c.9172delA	p.I3058fs*15	26.00	1472	25.00	1287	7.92	543	0.35	3442	
	TGFBR2	NM_003242.6	c.264G>A	p.W88*	22.00	1557	24.00	1123	8.20	500	ND		
	CHEK2	NM_001005735.2	c.1245_1246delinsTG	p.K416E	3.50	1692	ND		ND		ND		
	STK11	NM_000455.5	c.595G>A	p.E199K	1.13	1065	0.80	734	ND		ND		
	BAP1	NM_004656.4	c.878C>T	p.P293L	60.00	1550	57.00	1273	48.00	831	45	311	GV
	AR	NM_000044.6	c.475G>A	p.A159T	100.00	1028	100.00	934	99.00	353	100	170	GV
**32**	*TP53*	NM_000546.6	c.592G>T	p.E198*	0.28	3887			0.9	1167	1.83	1424	
**33**	*BRAF*	NM_004333.6	c.1799T>A	p.V600E	2.00	1502			0.39	4666	ND	2674	
	TP53	NM_000546.6	c.844C>T	p.R282W	1.28	1724			0.48	5445	ND	3670	
	SMARCD1	NM_139071.3	c.997G>T	p.E333*	4.16	1588			ND		ND	3360	
	ATM	NM_000051.4	c.1564_1565delGA	p.E522fs*43	46.00	1367			49.00	1522	49.00	1230	GV

	ATRX	NM_138270.5	c.5465A>G	p.N1822S	49.00	1207			51.00	1236	51.00	1078	GV
**51**	*KRAS*	NM_004985.5	c.35G>T	p.G12V	19.00	985			ND	2096	ND	3492	
	TP53	NM_000546.6	c.473G>T	p.R158L	8.15	932			ND	4715	ND	7421	

ND: Non detectable; GV: germline variant

*not detected initially by the analyze program until week 12. Results were manually analyzed retrospective. ** excluded due to change of treatment regime due to progression.

Supplementary Tabel S2: Dataset including therapy, outcome, and absolute values of all PET-parameters.

ID	Therapy	Outcome			SumSUL$_{peak}$			totalMTV$_{4.0}$			maxSUL$_{max}$			maxSUL$_{peak}$		
		Final response	OS (days)	PFS (days)	Baseline	Week 1	Week 3	Baseline	Week 1	Week 3	Baseline	Week 1	Week 3	Baseline	Week 1	Week 3
**2**	CT	PR	117	117	24.0	18.1	14.7	18.0	10.2	11.8	9.0	8.1	9.6	7.8	6.8	7.8
**6**	CT	PD	1042	85	34.6	39.3	30.8	11.4	11.0	11.4	24.2	35.4	19.9	17.6	22.1	15.6
**8**	CT	PR	580	267	28.3	34.3	18.7	39.3	40.5	23.6	23.2	27.0	13.5	16.2	18.7	9.1
**10**	CT	SD	1654	154	14.8	9.1	11.0	17.5	3.3	4.7	12.4	7.6	9.4	8.7	5.2	6.6
**11**	CT	PD	282	85	28.7	28.7	27.9	80.5	86.7	88.3	11.9	13.1	10.9	8.4	8.7	8.6
**12**	ICI	PD	145	47	31.4	31.4	23.1	26.6	26.7	28.8	13.7	13.5	13.9	10.3	10.1	10.9
**13**	CT	PR	593	455	8.1	5.5	2.6	11.8	2.5	0.1	12.5	8.1	4.5	8.1	5.5	2.6
**14**	ICI	PR	797	272	36.2	43.2	40.9	164.4	181.9	160.7	17.0	20.3	23.8	12.9	15.1	17.7
**22**	CT	SD	491	267	15.0		10.7	3.3		1.0	7.4		6.7	4.8		4.2
**26**	ICI	PD	90	90	21.8		24.3	9.6		16.9	11.7		10.1	5.6		7.1
**32**	CT	PD	88	53	16.4		29.1	22.3		34.4	11.6		19.5	7.3		10.0
**33**	ICI	PR	> 5 yrs	> 5 yrs	27.9		21.8	29.5		9.8	12.8		11.3	7.9		6.4
**40**	CT	PD	114	76	53.4		45.5	149.1		115.6	48.7		39.0	36.3		31.5
**51**	CT+ICI	PR	406	267	34.3		10.2	29.0		0.0	12.7		3.3	9.0		2.2
**52**	ICI	PD	231	86	12.2	14.0		86.4	95.7		18.6	18.3		12.2	14.0	
**53**	CT+ICI	PD	197	87	22.7		13.8	7.0		0.8	11.8		6.2	5.2		3.5

ID	meanSUL$_{mean50\%}$			meanSUL$_{mean4.0}$			totalMTV$_{50\%}$			totalTLG$_{50\%}$			totalTLG$_{4.0}$		
	**Baseline**	**Week 1**	**Week 3**	**Baseline**	**Week 1**	**Week 3**	**Baseline**	**Week 1**	**Week 3**	**Baseline**	**Week 1**	**Week 3**	**Baseline**	**Week 1**	**Week 3**
**2**	5.3	4.2	4.4	5.6	5.4	5.6	19.0	17.7	13.6	100.4	74.2	60.3	100.3	55.0	65.6
**6**	14.6	17.8	11.9	9.1	10.4	8.2	3.4	2.3	3.9	49.0	40.2	46.4	103.6	114.5	93.4
**8**	5.9	9.5	4.6	7.3	8.3	6.0	10.3	6.2	15.2	61.0	59.5	69.7	288.1	335.6	142.0
**10**	6.0	3.6	4.4	5.7	4.7	5.1	14.4	14.9	8.5	85.4	53.6	37.6	99.9	15.6	23.9
**11**	7.0	7.4	6.7	5.9	6.0	5.9	40.7	32.5	54.9	284.4	239.1	366.2	475.5	521.6	522.8
**12**	8.2	8.0	7.7	6.5	6.4	6.9	11.5	11.6	15.8	93.9	93.3	121.6	172.6	171.1	198.0
**13**	7.4	5.1	3.0	5.8	5.1	4.2	4.4	2.5	0.8	32.7	12.5	2.4	69.1	12.8	0.2
**14**	10.4	12.2	13.4	7.8	8.8	9.2	62.7	65.8	43.0	654.7	804.4	575.9	1283.6	1598.3	1478.3
**22**	3.5		3.3	4.9		4.8	8.7		5.2	30.9		17.2	16.2		4.8
**26**	4.7		5.1	5.0		5.2	11.9		15.6	55.4		79.3	48.0		87.4
**32**	6.9		10.4	5.8		6.9	11.0		6.3	75.7		65.5	129.7		238.6
**33**	6.6		5.3	6.0		5.3	17.2		7.4	112.4		38.7	176.2		51.3
**40**	25.9		21.9	12.0		11.9	20.4		24.0	527.0		526.4	1787.9		1371.9
**51**	7.1		1.9	6.1		0.0	16.7		20.0	118.6		37.4	177.4		0.0
**52**	10.9	11.5		7.3	7.7		15.4	24.3		168.5	278.0		629.3	732.2	
**53**	4.6		3.2	3.7		4.5	8.0		6.0	36.4		19.3	26.0		3.4

CT: chemotherapy; ICI: immunotherapy; NA: not available

Supplementary Table S3: Association between changes of PET-parameter (decrease vs. increase) and final response (PR+SD vs. PD).

Parameter	TN	FN	FP	TP	Accuracy
**Changes week 1**					
**ΔctDNA**	0	0	3	2	2/5
**Δ$maxSUL_{max}$**	3	2	2	2	5/9
**Δ$maxSUL_{peak}$**	3	1	2	3	6/9
**Δ$sumSUL_{peak}$**	3	2	2	2	5/9
**Δ$meanSUL_{mean50\%}$**	3	1	2	3	6/9
**Δ$meanSUL_{mean4.0}$**	3	1	2	3	6/9
**Δ$totalMTV_{50\%}$**	3	2	2	2	5/9
**Δ$totalMTV_{4.0}$**	3	1	2	3	6/9
**Δ$totalTLG_{50\%}$**	4	3	1	1	5/9
**Δ$totalTLG_{4.0}$**	3	1	2	3	6/9
**Changes week 3**					
**ΔctDNA**	5	1	0	2	7/8
**Δ$maxSUL_{max}$**	6	5	2	2	8/15
**Δ$maxSUL_{peak}$**	7	3	1	4	11/15
**Δ$sumSUL_{peak}$**	7	5	1	2	9/15
**Δ$meanSUL_{mean50\%}$**	7	5	1	2	9/15
**Δ$meanSUL_{mean4.0}$**	7	2	1	5	12/15
**Δ$totalMTV_{50\%}$**	6	2	2	5	11/15
**Δ$totalMTV_{4.0}$**	8	2	0	5	13/15
**Δ$totalTLG_{50\%}$**	7	4	1	3	10/15
**Δ$totalTLG_{4.0}$**	7	3	1	4	11/15

TN: true negative; FN: false negative; FP: false positive; TP: true positive; Δ = percentage change of the parameter; NA: not available
